# Epicardial adipose excision slows the progression of porcine coronary atherosclerosis

**DOI:** 10.1186/1749-8090-9-2

**Published:** 2014-01-03

**Authors:** Mikaela L McKenney, Kyle A Schultz, Jack H Boyd, James P Byrd, Mouhamad Alloosh, Shawn D Teague, Arturo A Arce-Esquivel, John N Fain, M Harold Laughlin, Harold S Sacks, Michael Sturek

**Affiliations:** 1 Departments of Cellular & Integrative Physiology, Indiana University School of Medicine, 635 Barnhill Drive, Room 385, Indianapolis, IN 46202-5120, USA; 2 Department of Cardiothoracic Surgery, Indiana University School of Medicine, Indianapolis, IN 46202, USA; 3 Department of Radiology, Indiana University School of Medicine, Indianapolis, IN 46202, USA; 4 Department of Biomedical Sciences, University of Missouri, Columbia, MO 65211, USA; 5 Department of Molecular Sciences, University of Tennessee Health Science Center, Memphis, TN 38163, USA; 6 Endocrinology and Diabetes Division, VA Greater Los Angeles Healthcare System, Los Angeles, CA 90073, USA; 7 David Geffen School of Medicine, UCLA, Los Angeles, CA 90095, USA

**Keywords:** Atherosclerosis, Computed tomography, Surgery, Intravascular ultrasound

## Abstract

**Background:**

In humans there is a positive association between epicardial adipose tissue (EAT) volume and coronary atherosclerosis (CAD) burden. We tested the hypothesis that EAT contributes locally to CAD in a pig model.

**Methods:**

Ossabaw miniature swine (n = 9) were fed an atherogenic diet for 6 months to produce CAD. A 15 mm length by 3–5 mm width coronary EAT (cEAT) resection was performed over the middle segment of the left anterior descending artery (LAD) 15 mm distal to the left main bifurcation. Pigs recovered for 3 months on atherogenic diet. Intravascular ultrasound (IVUS) was performed in the LAD to quantify atheroma immediately after adipectomy and was repeated after recovery before sacrifice. Coronary wall biopsies were stained immunohistochemically for atherosclerosis markers and cytokines and cEAT was assayed for atherosclerosis-related genes by RT-PCR. Total EAT volume was measured by non-contrast CT before each IVUS.

**Results:**

Circumferential plaque length increased (p < 0.05) in the proximal and distal LAD segments from baseline until sacrifice whereas plaque length in the middle LAD segment underneath the adipectomy site did not increase. T-cadherin, scavenger receptor A and adiponectin were reduced in the intramural middle LAD. Relative to control pigs without CAD, 11β-hydroxysteroid dehydrogenase (11βHSD-1), CCL19, CCL21, prostaglandin D_2_ synthase, gp91phox [NADPH oxidase], VEGF, VEGFGR1, and angiotensinogen mRNAs were up-regulated in cEAT. EAT volume increased over 3 months.

**Conclusion:**

In pigs used as their own controls, resection of cEAT decreased the progression of CAD, suggesting that cEAT may exacerbate coronary atherosclerosis.

## Background

In most recent reviews of human cross-sectional studies [1-3], the amount of epicardial adipose tissue (EAT), measured either as thickness by echocardiography over the right ventricle or as total volume by computed tomography (CT) scans, correlated directly with coronary artery disease (CAD) burden. Additionally, in prospective case-cohort [4] and in case–control [5] studies, EAT volume predicted future CAD events and myocardial ischemia. However, one report did not show that EAT volume added incremental predictive value over established CAD risk factors [6].

After regression analysis, EAT volume expansion in CAD occurs independently of associated increases in visceral abdominal and total body fat mass as well as changes in other CAD risk factors [2,3]. This epidemiological evidence together with pathophysiological data showing increased chronic inflammatory cell infiltrates [7] and up-regulation of pro-inflammatory, redox, macrophage marker, and angiogenic gene expression specifically in EAT sampled from humans undergoing coronary artery bypass for severe CAD [8] is the basis for the hypothesis that EAT might contribute locally and detrimentally to coronary atherogenesis. However, to our knowledge, there is no direct experimental evidence that EAT plays a role in coronary atherogenesis.

Our laboratory has characterized Ossabaw miniature swine as a model of CAD. The “thrifty genotype” of Ossabaw swine has enabled survival in the feast and famine ecology, i.e. extreme variations in food availability, of Ossabaw Island [9]. In the sedentary environment of captivity, our group induced CAD in this breed of pigs by feeding them a hypercaloric, atherogenic diet [9-12]. This animal’s coronary arterial supply more closely resembles that of humans compared to rodent research models. Additionally, rodent EAT is unpredictable in amount and not easily discerned, whereas the pig’s EAT is clearly demarcated macroscopically [9,11], can be imaged radiologically [9], and potentially might be amenable to surgical removal [13]. We tested the hypothesis that surgical excision of EAT in direct contact with the coronary artery (cEAT) of pigs would attenuate underlying plaque progression in vivo.

## Methods

### Animal model and protocol

We produced CAD in 9 castrated male Ossabaw miniature swine by feeding them 1000 g, once a day, of a high fat/cholesterol/fructose, atherogenic diet for 6 months starting at 6 months of age. The diet provided 16.3% kcal from protein, 40.8% kcal from complex carbohydrates, 19% kcal from fructose, and 42.9% kcal from fat. Fat calories derived from a mixture of lard, hydrogenated soybean oil, and hydrogenated coconut oil. It was supplemented with 2.0% cholesterol and 0.7% sodium cholate by weight (KT324, Purina Test Diet, Richmond, IN). Age- and gender-matched lean control swine were fed 750 g of regular chow (5 L80, Purina Test Diet, Richmond, IN) containing 18% kcal from protein, 71% kcal from complex carbohydrates, and 11% kcal from fat once a day. All animals had free access to drinking water. After 6 months on the diet, CT was performed to quantify EAT surrounding the heart. Surgical excision of cEAT covering the middle segment of the left anterior descending (LAD) artery, which we termed selective coronary adipectomy, was then conducted via a thoracotomy, as described below, followed immediately by intravascular ultrasound (IVUS) quantification of CAD. Following the procedure, the same baseline diet was continued during a 3-month recovery period at the end of which identical CT followed by IVUS were performed prior to sacrifice and tissue collection. Control pigs did not undergo IVUS or adipectomy because they have been shown previously to develop minimal or no CAD over the time course of this experiment [12]. In both adipectomy and control pigs at sacrifice, cEAT for mRNA analysis was resected from the adventitial surface of the LAD just proximal to the middle LAD adipectomy site, and as a comparator, paracardial fat was sampled from the mediastinal surface of the pericardium towards the apex of the heart. Samples were stored at −80°C. Biopsies of proximal, middle and distal LAD were taken and preserved for immunohistochemistry. All protocols involving animals were approved by an Institutional Animal Care and Use Committee and complied fully with recommendations in the *Guide for the Care and Use of Laboratory Animals*[14] and the American Veterinary Medical Association Panel on Euthanasia [15].

### Intravascular ultrasound

Following an overnight fast, swine received a 2.2 mg/kg of xylazine (Webster Veterinary, Devens, MA) and 5.5 mg/kg of telazol (Fort Dodge Animal Health, Fort Dodge, IA) intramuscular injection to induce anesthesia. Swine were intubated and anesthesia was maintained with 2-4% isoflurane in 100% O_2_ as a carrier gas. The isoflurane level was adjusted to maintain anesthesia with stable hemodynamics. Heart rate, aortic blood pressure, respiratory rate, and electrocardiographic data were continuously monitored throughout the procedure. A 7 F introducer sheath was inserted into the right femoral artery and heparin (200 U/kg) was administered under sterile conditions. A 7 F guiding catheter (Amplatz L, sizes 0.75-2.0; Cordis, Bridgewater, NJ) was advanced to engage the left main coronary ostium. A 3.2 F, 30 MHz IVUS catheter (Boston Scientific, Natick, MA) was advanced over a 0.014 inch diameter percutaneous transluminal coronary angioplasty guide wire (Boston Scientific, Natick, MA) and positioned in the LAD. Automated IVUS pullbacks were performed at 0.5 mm/sec in the LAD. Throughout the procedure angiography was performed to place the guiding and IVUS catheters in their desired positions. The IVUS catheter, guide catheter, and introducer sheath were removed and the right femoral artery ligated. The incision was closed and the animal was allowed to recover. Cefazolin (1000 mg; WG Critical Care, Lake Forest, IL) was given twice a day for six days following the procedure.

All IVUS pullback images were analyzed off-line (Windows Media Player, Microsoft) at 1 mm intervals. The LAD was divided into 3 segments: proximal (0–15 mm), middle (15–30 mm) and distal (30–45 mm) with 0 mm starting at the bifurcation of the left main into LAD and circumflex. Three IVUS pullback images were captured per vessel segment. Using the Image-Pro Plus software (Media Cybernetics, Rockville, MD), lumen area (LA) was found by tracing the vessel lumen. From LA, lumen circumference (LC) was calculated for each vessel segment by the following formula: LC = 2π [√ (LA/π)]. To derive wall coverage (WC) by plaque, the circumference of each cross-sectional image was divided into 16 equal segments. Percent wall coverage was calculated as (# segments containing atheroma ÷ 16) × 100. From the product of average WC × LC, a circumferential plaque length for each vessel segment was found. As an example, the following results are presented from a proximal coronary artery segment:

LA 21.40 mm^2^; LC 16.40 mm; WC 29.58%; circumferential plaque length = 4.85 mm. Pullbacks were used for 7 out of 9 animals due to restrictions from vasospasms or lumen diameter. Using a 2-way analysis of variance (ANOVA), average circumferential plaque length per vessel segment was compared pre- and post-adipectomy.

### Selective adipectomy

**Figure 1:**
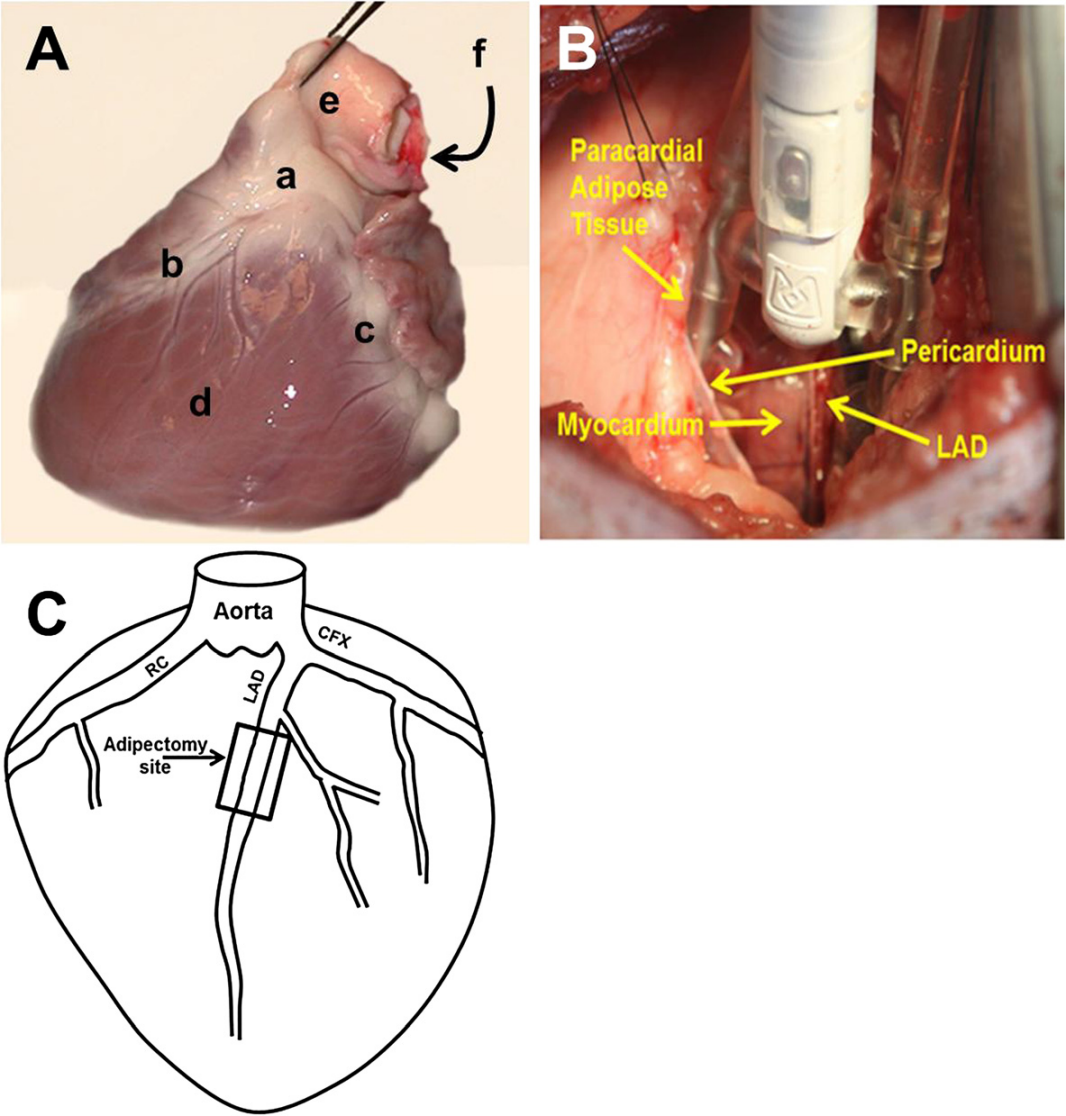
**Ossabaw swine heart anatomy and adipectomy procedure. A**. Ossabaw heart (left anterior view) ex vivo. a. epicardial adipose tissue; b. left anterior descending artery; c. circumflex artery; d. left ventricle; e. pulmonary artery; f. aorta. **B.** Adipectomy procedure with Octopus stabilizing the myocardium and left anterior descending (LAD) segment. **C**. Surgical landmarks for excision of epicardial adipose tissue; RC, right coronary artery; LAD, left anterior descending artery; CFX; circumflex artery.

Under general anesthesia, cardiovascular monitoring (both as described above), and using aseptic surgical techniques, all adipectomies were performed on the LAD with access via left thoracotomy by the cardiac surgeon. Figure 1A shows the anatomical structures and distribution of EAT over the epicardium of a representative pig heart ex vivo. A Thoratrak Retractor (Medtronic; Minneapolis, MN) was utilized for minimal rib spreading and as a base for the Octopus cardiac stabilizer (Medtronic; Minneapolis, MN) (Figure 1B). The pericardium was opened and cradled to the drapes exposing the LAD. The Octopus was then positioned to stabilize the middle LAD segment. Using the bifurcation of the left main into LAD and circumflex as the reference point, cEAT was excised sharply starting 15 mm distal to this landmark for a distance of 15 mm along the length of the LAD and 3–5 mm on both the right and left lateral aspects of this part of the vessel (Figure 1C). Non-absorbable Prolene sutures were placed to control epicardial venous bleeding from resected fat typically to the left side of the LAD. The ligatures demarcated this site for later tissue collection at sacrifice. When possible, the pericardium was loosely approximated with interrupted sutures. The chest was then closed in layers in the standard fashion. Pleural air was evacuated by means of suction on an intrapleural red rubber catheter being withdrawn during a valsalva breath and closure of the chest wall musculature. One of the 9 animals succumbed intra-operatively from an acute myocardial infarction complicated by refractory ventricular fibrillation documented by ECG and by subsequent autopsy that showed acute LAD thrombosis. There were no noticed post-operative complications in the remaining swine.

### Immunohistochemistry

Sections of the LAD were prepared using techniques previously described by us [16]. Samples of LAD were dissected and immersed in neutral-buffered 10% formalin for a minimum of 24 h and then processed to paraffin embedment. The proximal, middle, and distal samples of LAD were obtained consistently from the same location in all pigs. Five-micrometer sections were cut with an automated microtome (Microm, Thermo Fischer Scientific, Bellefonte, PA), floated onto positively charged slides (Thermo Fischer Scientific), and deparaffinized. The slides were then steamed in citrate buffer at pH 6.0 (Dako target retrieval solution S1699; Dako, Carpenteria, CA) for 30 min to achieve antigen retrieval and subsequently cooled for 30 min. Next, the slides were stained manually with sequential Tris buffer or water wash steps performed after each protocol step. Sections were incubated with avidin biotin two step blocking solution (Vector SP-2001; Vector Laboratories, Burlingame, CA) to inhibit background staining and in 3% hydrogen peroxide to inhibit endogenous peroxidase. Nonserum protein block (Dako X909; Dako) was applied to inhibit nonspecific protein binding. Immunostaining was performed using scavenger receptor A (SRA-ES; 1:100 dilution, Transgenic Inc. #KT-022), tryptase (1:600 dilution, Dako #M7052), CD-3 (1:600 dilution, Dako #A0452), monocyte chemoattractant protein-1 (MCP-1; 1:100 dilution, PeproTech #500-P76), resistin (1:800 dilution, Phoenix Pharmaceuticals #H-02840), T-cadherin (1:1000 dilution, Abgent #CDH13, AP14346), adiponectin (1:200, Chemicon #MAB 3604) or tumor necrosis factor-alpha (TNF-alpha; 1:100 dilution, R&D #AF690). All the primary antibodies were diluted using antibody diluent (S0809; Dako) and were incubated with the tissue sections overnight at 4-C. After the appropriate washing was completed, sections were incubated with biotinylated antimouse or rabbit link secondary antibody and then peroxidase-labeled streptavidin (LSAB + kit, peroxidase, K0690; Dako). Diaminobenzidine (K3468; Dako) was applied for 5 min and allowed visualization of primary antibody staining. Sections were counterstained with Mayer hematoxylin for 1 min, dehydrated, and coverslipped. For negative controls, histological sections were prepared as described, but incubation in primary antibody was replaced with Tris buffer.

Immunostaining analysis was performed in the entire vessel wall (i.e. intima to external elastic lamina boundaries) in order to quantify the presence of SRA, tryptase, CD3, MCP-1, resistin, T-cadherin, adiponectin or TNF-alpha. Sections were examined using an Olympus BX61 photomicroscope (Olympus, Melville, NY), and pictures were captured at 20x magnification. Image-Pro Plus (version 6.2.0.424; Media Cybernetics, Inc., Silver Spring, MD) was used to identify and quantify the positive area of staining. Percent staining of the vessel wall was calculated from the data for positive staining and total area. An experienced investigator that was blinded to treatment group’s identities performed the selection of target areas, photography, and image analysis.

### mRNA analysis

#### mRNA Isolation

Approximately 0.2 to 0.5 g of frozen cEAT and paracardial fat from adipectomy and control animals was homogenized with 5 ml of a monophasic solution of phenol and guanidine isothiocyanate (TRIzol reagent, Invitrogen, Carlsbad, CA).

#### RT-PCR

The mRNA assay involved real-time quantitative PCR. Transcriptor First Strand cDNA synthesis Kit from Roche Diagnostics was used on equal quantities of RNA to prepare the complementary DNA (cDNA). The Roche Lightcycler 480 Real-time RT-PCR system and Roche’s Universal Probe Library of short hydrolysis Locked Nucleic Acid (LNA) dual hybridization probes combined with the primers recommended by their web-based assay design center [http://www.universalprobelibrary.com] were used for mRNA quantification. Integrated DNA Technologies (Coralville, IA) synthesized the primers. Twenty five genes related to atherosclerosis were selected (Table 1). Fifteen mRNAs were targeted for their roles in inflammation, 4 for their roles in oxidative stress as well as reactive oxygen species regulation and 6 in control of angiogenesis and endothelial cell function. In each assay, 55 ng per tube of total RNA (determined by absorption at 260 nm in a nano spectrophotometer) was used. The data were obtained as crossing point values (Cp) obtained by the second derivative maximum procedure as described by Roche Applied Science technical notes LC10/2000 and 13/2001 [http://www.roche-applied-science.com/sis/rtpcr/htc/index.jsp]. Relative quantification of the data was calculated using the absolute Cp values based on analyzing the same amount of total RNA in each assay as recommended by Bustin [17].

**Table 1:** Ratio of gene expression in fat from obese and lean Ossabaw pigs

	**Ratio of obese/lean**	
**mRNA**	**Epicardial Fat**	**Paracardial Fat**
Inflammatory adipokines or proteins associated with inflammation		
11β HSD-1	16.0#	1.2
CCL19	14.0#	4.4*
Adiponectin	6.7***	5.0*
Prostaglandin D_2_ synthase	5.3#	3.0**
CCL21	3.1*	1.0
TLR4	1.8	2.0**
IL-6	1.7	1.8
NGF	1.6	1.6
IL-8	1.6	1.4
Unc5b	1.4	1.1
Netrin-1	1.3	0.6
CCR7	1.2	0.8
TNF	1.1	1.7
IL-1β	0.8	0.6
Leptin	0.7	1.5
Oxidative stress/reactive oxygen species regulation		
gp91phox [NADPH oxidase]	72.0#	5.4***
SOD	2.0	1.5
cytochrome C oxidase	1.5	1.5
cyclooxygenase 2	2.2	1.1
Angiogenesis and endothelial cell function		
VEGFa	16.0#	4.3***
Angiotensinogen	5.3#	1.6
VEGFGR1	4.3**	4.3***
Endothelin-1	2.5	1.7
eNOS	1.1	1.4
adenosine receptor 1	0.7	0.9

**Legend:** mRNA ratios of obese (n = 8) to lean (n = 8) swine. Significant values are depicted as follows: * 0.05, ** 0.025, *** 0.01, # 0.001.

### Computed tomography

**Figure 2:**
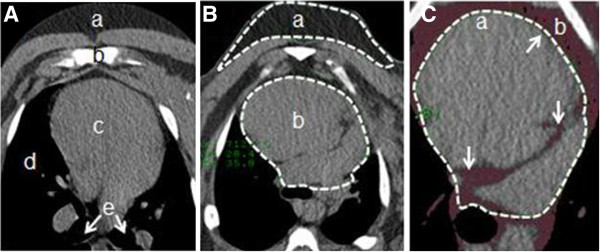
**Epicardial fat CT imaging.** Transverse slices were taken at the level of the carina, approximately midway between the base and apex of the heart. **A**. Landmark CT: a. subcutaneous adipose tissue; b. sternum; c**.** heart; d. lungs; e. bronchi. **B**. Representative ROIs: a. subcutaneous reference ROI; b. ROI drawn to encapsulate pericardium (fat inside ROI considered epicardial fat); **C**. CT image after fat analysis (maroon = fat); a. ROI arrows drawn to show epicardial fat locations; b. paracardial fat. CT = computed tomography, ROI = region of interest.

CT scans to quantify changes in EAT were analyzed by one observer. EAT was defined as fat between the inner surface of the visceral pericardium and epicardial surface of the myocardium [18,19]. Paracardial fat was defined as fat lying within the mediastinum superficial to the parietal pericardium [18,19]. Representative images are shown in Figures 2A, B and C. The pigs were scanned using a Philips Brilliance 64 detector scanner (Cleveland, OH) while sedated using 4% Isoflurane (Webster Veterinary, Devens, MA) supplied by mask. Imaging was performed with retrospective electrocardiogram (ECG) gating and without the administration of intravenous contrast from above the aortic arch to below the heart. Additional scan parameters included a 64 × 0.625 collimation, pitch of 0.2, rotation time of 0.4 seconds, 120 kVp, and 800–1000 mAs. Images were reconstructed with a slice thickness of 0.67 mm and increment of 0.67 mm using a CB filter. A batch was first created to ensure equal amounts of data were analyzed and included for each image set. The baseline and pre-sacrifice images were then aligned side-by-side to ensure similar magnification and orientation. Transverse images were analyzed by starting at the superior aspect of the atrial appendage. Sternal, vertebral body and cardinal landmarks were utilized to insure similar alignment. The slice thickness of each image produced was 3 mm without overlap, yielding about 40 images per heart in each batch. Regions of interest (ROI) were drawn by hand using quantitative computer tomography angiography (Q-CTA) software on a Philips Extended Brilliance Workspace (Version 3.5) to include the entire heart. Vessels leaving and entering the heart were included until they were noticeably detached from the heart. The Hounsfield unit (HU) value is similar for blood, muscle, and soft tissue, thus making the vessels appear as part of the heart until a visible distance is evident between the heart and the vessel. In all cases vessels were split in the ROI through their center as the pericardium encapsulates the most proximal regions of the vessels leaving and entering the heart. The software highlighted all tissue that matched the HU intensity of a reference ROI drawn in known subcutaneous fat (at the level of the carina). The fat area measurements of the images were then multiplied by their slice thickness to determine fat volume. Volumes of EAT were compared before adipectomy and at the end of the recovery period. Inter-operator coefficient of variation was 6.2%, while intra-operator variation was 1.0%.

### Statistics

Statistical analysis was performed via Microsoft Excel 2010, utilizing ANOVA, regression analysis and post hoc 1- and 2-tailed paired student t-tests to examine significance. All values are given as mean ± SE. A p value <0.05 was considered statistically significant.

## Results

### Metabolic characteristics

At adipectomy, pigs on the atherogenic diet had developed CAD and its risk factors including hypertension, obesity, increased LDL/HDL ratio, insulin resistance, glucose intolerance and increased triglyceride levels compared to age-matched lean controls (Table 2). Other than the increase in body weight, these characteristics remained constant during the 3 month recovery following adipectomy (data not shown).

**Table 2:** Metabolic characteristics of Ossabaw swine

**Parameter**	**Obese (n = 9)**	**Lean (n = 9)**	**P value**
Weight at adipectomy (kg)	77 ± 3	N/A	N/A
Weight at sacrifice (kg)	91 ± 1	54 ± 2	< 0.001
Total cholesterol (mg/dL)	576 ± 40	57 ± 6	< 0.001
Fasting triglycerides (mg/dL)	108 ± 21	32 ± 3	< 0.001
LDL (mg/dL)	502 ± 36	33 ± 1	< 0.001
HDL (mg/dL)	53 ± 7	24 ± 2	< 0.005
LDL/HDL ratio	11 ± 2	1 ± 0.1	< 0.001
Fasting glucose (mg/dL)	80 ± 5	73 ± 6	0.334
Plasma glucose peak (mg/dL)	820 ± 35	527 ± 24	< 0.001
Plasma glucose AUC	17138 ± 1004	9274 ± 377	< 0.001
Fasting insulin (μU/ml)	21 ± 6	5 ± 1	0.020
Plasma insulin peak (μU/ml)	172 ± 19	33 ± 4	< 0.001
Plasma insulin AUC	4413 ± 432	667 ± 121	< 0.001
Systolic blood pressure (mmHg)	138 ± 2	110 ± 3	<0.001
Diastolic blood pressure (mmHg)	79 ± 2	67 ± 2	<0.030

**Legend:** Adipectomised obese swine were age matched to lean swine. Values for adipectomised swine were obtained just prior surgery (n = 9), except for body weight at sacrifice (n = 8). AUC = area under curve, HDL = high density lipoprotein, LDL = low density lipoprotein.

### IVUS

**Figure 3:**
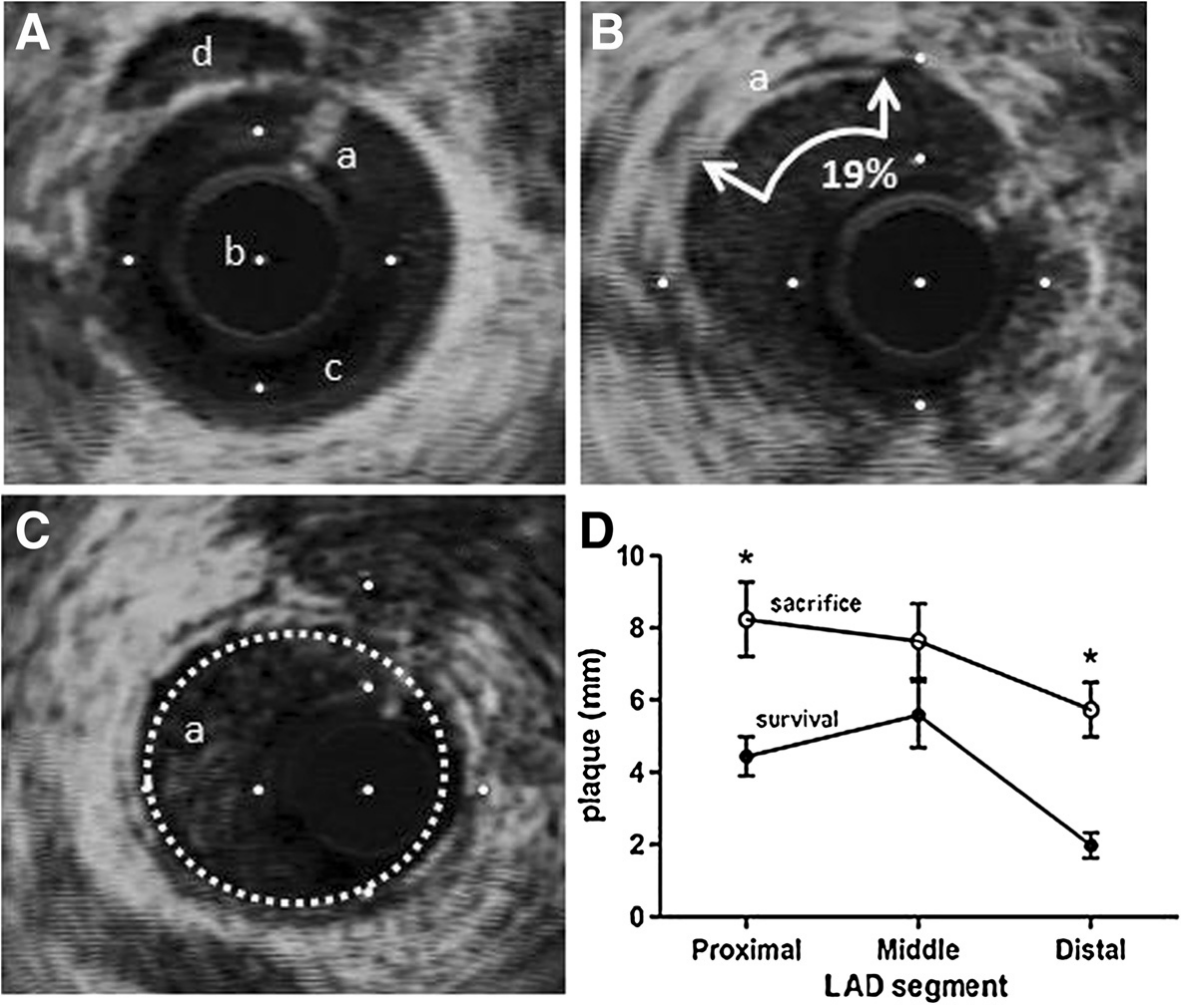
**IVUS images and analysis. A-C:** IVUS images, distance between white dots =1.0 mm. **A**. Normal vessel with no intimal thickening; a. guidewire artifact b. IVUS catheter c. clear lumen d. lumen of parallel vessel. **B**. Analysis example; a. atheroma covering 3 segments of a 16 segment designation ~ 19%. **C**. a. Concentric neointimal ring of atheroma. **D**. Circumferential plaque length in each 15 mm of proximal, middle and distal left anterior descending coronary artery (LAD) at the time of adipectomy (survival, lower line) and at sacrifice (3 months later, upper line). Values are mean ± sem for 7 pigs. Asterisk indicates p < 0.05 between upper and lower proximal and distal LAD portions.

Representative IVUS images in Figure 3 show a normal coronary lumen (A) as well as partial (B) and complete circumferential (C) neointimal plaque formation. The results of IVUS pullbacks revealing atherosclerosis in each 15 mm length proximal, middle and distal portions of the LAD from its origin are shown in Figure 3D. Over 3 months, baseline mean circumferential plaque length in the proximal LAD increased from 4.45 ± 0.54 to 8.24 ± 1.03 mm (p < 0.01, Two-way ANOVA and Bonferroni post-hoc) and in the distal LAD from 1.98 ± 0.35 to 5.74 ± 0.75 mm (p < 0.01, Two-way ANOVA and Bonferroni post-hoc). By contrast, circumferential plaque length in the middle LAD increased from 5.60 ± 0.90 to 7.64 ± 1.03 mm which was not statistically different (p > 0.05, Two-way ANOVA and Bonferroni post-hoc). There was no difference between baseline mean proximal and middle LAD plaque lengths (p = 0.26), whereas middle LAD was higher than distal LAD (p = 0.01) and proximal was also greater than distal (p = 0.04).

### Immunohistochemistry

**Figure 4:**
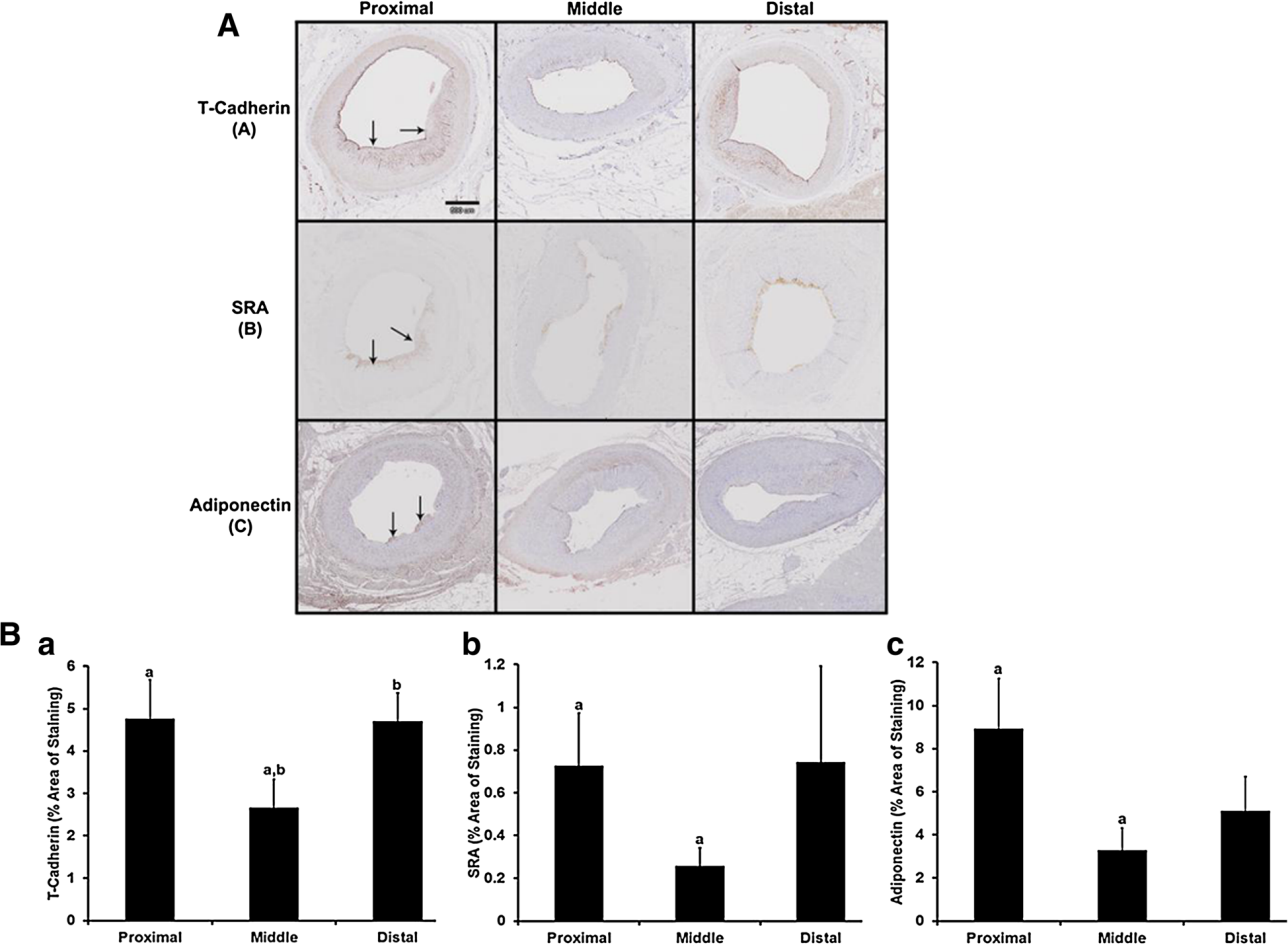
**Immunohistochemistry of proximal, middle, and distal LAD. A:** Representative photomicrographs of brown immunostaining for T-Cadherin [A, upper panels], SRA [B, middle panels] and Adiponectin [C, lower panels] in the designated LAD segments. Arrows point to brown positive stains in the endothelial and sub-endothelial neo-intima for each marker. Scale bar = 500 microns. **B:** Percent immunohistochemical staining of the proximal, middle and distal 15 mm segments of left anterior descending coronary wall intima-media for a; T-Cadherin, b; SRA and c; adiponectin. The same letters (a-a, b-b) indicate that the mean ± sem in the segments are significantly different (n = 8 separate samples from each segment, p < 0.05).

On light microscopy of the LAD middle segment sections, a thinner layer of cEAT covered the adventitia than the adjacent two segments, suggesting no regrowth of remnant fat 3 months after adipectomy. Figure 4A shows representative photomicrographs of the presence, distribution and intensity of immunostaining of T-cadherin, SRA and adiponectin in the three segments of the LAD. T-Cadherin was strongly positive in the endothelial layer, but also present in the sub-endothelial neointima in proximal and distal LAD, but to a lesser extent in the middle vessel wall. SRA was present in the endothelial layer and neointima. Adiponectin was present in the endothelial layer and very dispersed over the media. As shown in Figure 4B (panel A), when these markers in all samples were quantitated, there was a significant reduction in mean percentage area of T-cadherin staining in the intima-media from 4.77 ± 0.92% in the proximal and 4.71 ± 0.66% in the distal LAD segments to 2.67 ± 0.66% in the middle LAD (proximal to middle LAD p = 0.047 and distal to middle LAD p = 0.01 using 2-tailed t tests). SRA staining (Figure 4B, panel B) was 0.73 ± 0.25% in the proximal LAD and 0.26 ± 0.08% in the middle LAD which was significantly different (p = 0.035) by a 1-tail t test with a trend towards significance (p = 0.07 in a 2-tailed t test), and 0.74 ± 0.45% in the distal LAD which although higher than the middle LAD was not significantly different. Adiponectin staining (Figure 4B, panel C) was significantly lower in the middle LAD 3.28 ± 1.04% versus the proximal 8.94 ± 2.32% (p = 0.04 in a 1-tailed t test versus a trend for significance p = 0.08 in a 2-tailed t test). In the distal LAD, adiponectin measured 5.12 ± 1.57% which was not different to either the proximal or middle LAD. There were no differences in coronary wall per cent staining for tryptase, CD3, TNFα, MCP-1 and resistin between the proximal, middle and distal LAD.

### CT quantification of epicardial fat

**Figure 5:**
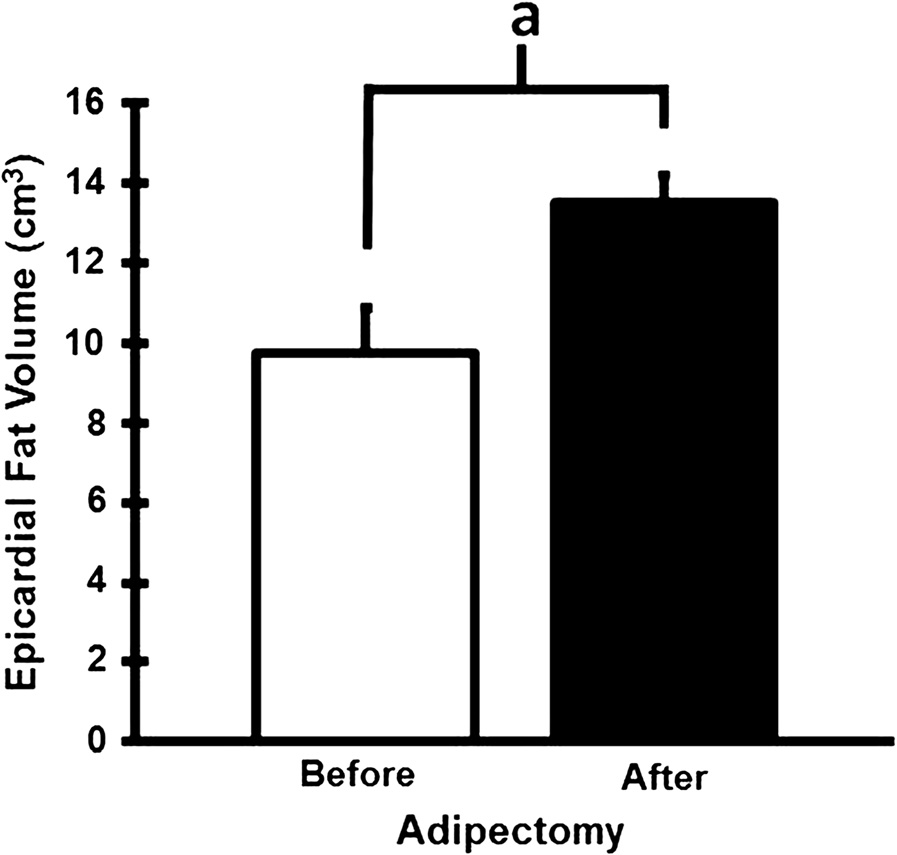
**Epicardial adipose tissue volumes of swine on high-fat diet before adipectomy and 3 months after the procedure.** Letter a indicates significant difference, p < 0.01.

In Figure 5, CT scans done on 6 of the 8 surviving swine showed an increase in total EAT volume from 9.7 ± 1.1 cm^3^ at adipectomy to 13.5 ± 0.7 cm^3^ at time of sacrifice, representing a 39% increase (p = 0.006) over 3 months. Due to inadequate numbers of data points, CT data could not be correlated with the IVUS data to determine if a relationship existed between changes in EAT volume and total plaque burden.

### Gene expression

Table 1 shows the expression in cEAT and paracardial fat of genes linked to atherogenesis, namely those involved in inflammation, reactive oxygen species and redox reactions, angiogenesis and endothelial cell function. The data in each fat depot are expressed as the ratio of experimental to control pig fat depots. In EAT, there were significant and substantial fold increases in mRNA expression of pro-inflammatory 11β-hydroxysteroid dehydrogenase (11βHSD-1), CCL19, CCL21 and PGD2S; anti-inflammatory adiponectin; pro-oxidant gp91phox [NADPH oxidase], angiogenic VEGF and VEGFGR1 and vascoconstrictive angiotensinogen. There were no changes in pro-inflammatory toll-like receptor 4 [TLR4], CCR7, TNFα, IL-1β, IL-6, IL-8, leptin, Unc5b, netrin-1 and NGF; redox genes eNOS, superoxide dismutase [SOD], cyclooxygenase 2 and cytochrome oxidase; and vasoconstrictor endothelin-1 and vasodilator adenosine receptor 1. By contrast, in paracardial fat relative to cEAT, 11βHSD-1 and CCL21 were normal; CCL19, PGD2S, adiponectin pro-oxidant gp91phox [NADPH oxidase] VEGF, VEGFGR1 and angiotensinogen were up-regulated to much lesser degrees; and the remaining genes were similar except for TLR4 and endothelin-1 which were modestly (~2 fold) increased.

## Discussion

We present experimental evidence that, in Ossabaw miniature swine, selective surgical excision of adipose tissue in direct contiguity with one of the epicardial coronary arteries attenuated the progression of atherosclerosis, thus supporting the hypothesis that cEAT could contribute to underlying coronary atherogenesis [13]. The findings are applicable to the early stages of CAD because of the relatively young age of the animals, the short duration of atherogenic diet feeding, and the lack of observed flow-limiting coronary stenosis typical of advanced clinical disease. We acknowledge the substantially high LDL cholesterol levels (>500 m/dL) in the obese group. We have conducted other studies in which the LDL was ~250 mg/dL (e.g. [10,12]) for a longer duration, which yielded substantial coronary atherosclerosis. We predict a similar result of adipectomy. This very high LDL cholesterol level for a short duration is similar to LDLR−/− humans, who have substantial atherosclerosis. Horton and colleagues refer to this phenomenon as the effect of cumulative LDL cholesterol in g/dL-years [20].

We showed that in the middle LAD at the adipectomy site, progression of CAD was not significant compared to the significant increases in circumferential plaque in the adjacent, unperturbed proximal and distal segments. Thus, in the middle LAD segment, the increase in mean plaque length was 1.4-fold compared to 1.9- and 2.9-fold in the proximal and distal LAD, respectively. There were higher mean values and greater variability in baseline plaque length in the middle compared to the proximal and distal segments at the time of adipectomy, suggesting that the attenuated progression of atheroma could have been the consequence of higher initial plaque thickness at the inception of the protocol. However, a two-tailed, paired t-test did not indicate that this baseline difference was statistically significant (p = 0.26). The reason for the variability in the middle LAD plaque dimensions is not clear, since prior studies have indicated that mean plaque burden is greatest proximally and decreases distally in the LAD [12]. One possibility is that adipectomy could have perturbed underlying plaque structure or vasomotor responses and interfered with IVUS measurements performed under heparin immediately after adipectomy. However, we have recently shown that cEAT has uniformly adverse effects on coronary artery function [21], in contrast to protective effect of PVAT in the aorta and other arteries. This explains why we observed no adverse mechanical changes after adipectomy.

We resected fat from one coronary artery for several reasons. Resection of as much fat as possible off the entire myocardium was not technically possible in vivo. cEAT on the LAD was the most accessible site within the operative field of exposure compared to either the circumflex or right coronary arteries, which would lessen both intraoperative trauma, morbidity and mortality, and the risk of post-operative complications. This strategy targeting one rather than two vessels was subsequently vindicated by the low intraoperative mortality (1 of 9 pigs) and no noticed post-operative complications. Finally, the longitudinal time course of the experiment enabled the pigs to act as their own controls. Immunoreactivity for T-cadherin was predominantly localized to the endothelium as previously established [22], but also to the neo-intima. It is considered to be a marker of active atherosclerosis [22] and a major receptor for adiponectin in the vasculature [23]. T-cadherin and SRA, the scavenger receptor for LDL-cholesterol on plasma membranes of plaque macrophages and foam cells [24,25], were reduced in the middle LAD intima-media implying less atherogenesis. Paradoxically, anti-inflammatory immunoreactive adiponectin in the same LAD segment was reduced significantly by 68%, despite a ~7-fold upregulation of adiponectin mRNA expression in overlying cEAT; thus, one might surmise that adiponectin released by epicardial adipocytes might not have been able to penetrate the coronary wall if such a mechanism exists. While a majority of the literature describes a decrease in adiponectin expression in CAD patients, we speculate that the 6.7-fold increase in obese pig cEAT mRNA was a compensatory response and that there must be some block in mRNA translation. We have previously reported that serum adiponectin is decreased in obese versus lean Ossabaw swine [26]. On the other hand, there were no changes in vessel wall CD3 (a T lymphocyte cell marker), tryptase (a basophil marker), TNFα, MCP-1, and resistin.

The mechanisms whereby selective adipectomy might have attenuated the progression of CAD remain to be established, but hypothetically include (i) the removal of factors generated by cEAT that could contribute to atherogenesis by direct diffusion through the porous adventitia into the coronary intima-media [19], such as reactive oxygen species (ROS) and pro-inflammatory cytokines and/or (ii) the disruption of vasa vasorum in the adventitia and in the closely adjacent cEAT [27], which might interfere with vasocrine signaling of atherogenic adipokines [19] and restrict mononuclear cell access into the intima-media. The evidence for these suppositions includes the fact that relative to control cEAT, there was significant up-regulation in atherosclerotic cEAT of pro-inflammatory 11βHSD-1, CCL19, CCL21 and PGD2S; gp91phox [NADPH oxidase], a major source of vascular ROS; VEGF and VEGFGR1, which partake in vasa vasorum neogenesis and angiotensinogen, which promotes endothelial dysfunction and vasoconstriction. Similar to our current findings in pigs, we reported 3-fold up-regulation of 11βHSD-1 gene expression in human EAT adjacent to severe CAD over controls without CAD [8]. Pharmacological inhibition of 11βHSD-1 prevented aortic plaque progression in a murine model of atherosclerosis, suggesting that 11βHSD-1 may play a role in atherogenesis [28]. Lymphoid chemokines CCL19 and CCL21 are crucial for the recruitment of circulating naive T cells into lymph nodes. Increased levels of CCL19 and CCL21 have been reported within the atherosclerotic lesions of ApoE−/− mice, in human atherosclerotic carotid plaques, and in plasma of CAD patients [28]. In mice, PGD2S deficiency induces obesity and facilitates aortic atherosclerosis [29], suggesting it is normally protective and anti-atherogenic in this species. The role of PGD2S in porcine atherosclerosis has not been reported, but -regulation of PGD2S in EAT in our study was associated with amelioration of atherosclerosis, suggesting the possibility that this pro-inflammatory prostanoid might otherwise be harmful either alone or in conjunction with other adipokines. No changes were observed in cEAT in the expression of TLR4 and acute phase cytokines TNFα, IL-1β, IL-6, IL-8 as well as the anti-oxidant genes eNOS and SOD. Finally, the enhanced expression of a selected array of pro-atherosclerotic genes in cEAT over paracardial fat attests to the relative specificity of the genomic response of cEAT associated with CAD that has been similarly documented in humans with CAD [8].

Epicardial fat volume determined by sequential CT scans significantly increased over 3 months of hypercaloric feeding under the same conditions previously shown to result in major expansion of visceral retroperitoneal and intraperitoneal as well as subcutaneous abdominal fat depots in the same animal model [30]. Although our experimental design using pigs as their own controls in a longitudinal study has its strengths, there are also limitations in the lack of sham-adipectomies in age- and gender-matched pigs fed hypercaloric atherogenic diet. Future studies should employ sham-operated animals; randomization of experimental and control pigs; blinding of both the ultrasonographer and two independent IVUS data analysts to the pig group designation; reversing heparin anti-coagulation during IVUS with protamine sulphate so that IVUS can be done before surgery; CT scanning using radio-contrast dye to delineate the coronaries and to quantitate cEAT area around coronary plaques per se rather than EAT covering the entire myocardium; and immunohistochemical and mRNA analysis of cEAT and the coronary wall for expression of genes and proteins known to be important mediators of or participants in atherogenesis. To make these studies more translatable to clinical treatment, more clinical outcomes studies in the swine model would be helpful, for example, monitoring hard clinical endpoints such as MI, fatal events, etc. over a longer duration.

Our translational research project was done using experimental conditions prohibited in man by obvious ethical constraints and, with the provisos noted above, supports the human epidemiological evidence for a direct relationship between EAT and CAD. At this juncture and until more rigorous evidence is available one way or the other, it is premature to advocate an adipectomy as surgical treatment to attenuate the progression of CAD. Rather, it would be more appropriate from a clinical point of view if non-invasive methods were devised to shrink epicardial fat volume to serve the same beneficial purpose. In this regard, weight loss by caloric restriction and/or exercise in obese human subjects is associated with shrinkage of EAT volume by 9-32% of baseline in different reports [13,31], but it is not known whether this is accompanied by decreases in chronic inflammation in EAT or reduction in CAD burden [12].

## Conclusions

The results of our pilot study are consistent with the hypothesis that selective coronary artery adipectomy attenuates the progression of early atherosclerosis, setting the stage for performing definitive future studies using sham operation controls. These studies will determine whether coronary adipectomy attenuates stable atherosclerotic plaques typically found in clinically advanced disease.

## Abbreviations

CAD: Coronary artery disease; cEAT: coronary epicardial adipose tissue; CT: Computed tomography; EAT: Epicardial adipose tissue; ECG: Electrocardiogram; F: French (length); HDL: High density lipoprotein; HU: Hounsfield Unit; IVUS: Intravascular ultrasound; LAD: Left anterior descending coronary artery; LDL: Low density lipoprotein; mEAT: myocardial epicardial adipose tissue; ROI: Region of interest; Q-CTA: Quantitative computed tomography angiography.

## Competing interests

The authors declare that they have no competing interests.

## Authors’ contributions

MLM analyzed the IVUS data, coordinated the work and wrote/edited the manuscript; KAS performed the CT studies, coordinated the work and wrote/edited the manuscript; JHB performed the adipectomies; JPB and MA provided technical assistance and edited the manuscript; SDT performed and supervised CT scans; AAA-E performed Immunohistochemistry; JNF assayed the genes by RT-PCR; MHL reviewed and edited the manuscript; HSS and MS devised the experiments, reviewed and edited the manuscript. All authors read and approved the final manuscript.
